# Influence of population density, temperature, and absolute humidity on spread and decay durations of COVID-19: A comparative study of scenarios in China, England, Germany, and Japan

**DOI:** 10.1016/j.onehlt.2020.100203

**Published:** 2020-12-11

**Authors:** Yinliang Diao, Sachiko Kodera, Daisuke Anzai, Jose Gomez-Tames, Essam A. Rashed, Akimasa Hirata

**Affiliations:** a Department of Electrical and Mechanical Engineering, Nagoya Institute of Technology, Nagoya 466-8555, Japan; b Center of Biomedical Physics and Information Technology, Nagoya Institute of Technology, Nagoya 466-8555, Japan; c Department of Mathematics, Faculty of Science, Suez Canal University, Ismailia 41522, Egypt

**Keywords:** COVID-19, Temperature, Absolute humidity, Population density, Multivariable analysis

## Abstract

In this study, we analyzed the spread and decay durations of the COVID-19 pandemic in several cities of China, England, Germany, and Japan, where the first wave has undergone decay. Differences in medical and health insurance systems, as well as in regional policies incommoded the comparison of the spread and decay in different cities and countries. The spread and decay durations in the cities of the four studied countries were reordered and calculated based on an asymmetric bell-shaped model. We acquired the values of the ambient temperature, absolute humidity, and population density to perform multivariable analysis. We found a significant correlation (*p* < 0.05) of the spread and decay durations with population density in the four analyzed countries. Specifically, spread duration showed a high correlation with population density and absolute humidity (*p* < 0.05), whereas decay duration demonstrated the highest correlation with population density, absolute humidity, and maximum temperature (*p* < 0.05). The effect of population density was almost nonexistent in China because of the implemented strict lockdown. Our findings will be useful in policy setting and governmental actions in the next pandemic, as well as in the next waves of COVID-19.

## 1 Introduction

The outbreak of the coronavirus disease (COVID-19) has been reported worldwide, and it has reached more than 200 countries [1,2]. Numerous studies on COVID-19 have investigated meteorological factors, clinical factors, and public health interventions that affect infection and morbidity [[3], [4], [5], [6]]. Commonly, the morbidity and mortality rates of COVID-19 differ by one or more orders of magnitude in each country. Some hypotheses for this observation have been presented but remain controversial, such as the effects of the Bacillus Calmette–Guérin vaccine [7,8], age structure [9,10], and race [11,12].

Another factor that policies should consider is the duration of the lockdown [[13], [14], [15]]. However, proper comparison is not straightforward because of various cofactors, such as human behavior and regional policies; a similar problem occurs with the determination of the morbidity and mortality rates.

During the constraint, the public was stressed because of the lack of information on potential risk factors. Several restrictions negatively impact different life aspects, including employment rate and education progress. Therefore, it is important to determine which social and environmental factors may influence the morbidity and mortality rates in different societies. Thus, the analysis of COVID-19 data will ensure the appropriate policy setting and governmental actions during the pandemic. The establishment of effective policies is closely related to the available medical resources and the required duration of restrictions (e.g., city lockdown). Different lockdown and restriction measures have been applied worldwide. A summary of the lockdown policies in China, England, Germany, and Japan is listed in Appendix.

Among the other cofactors, ambient temperature and humidity have been extensively investigated worldwide [16,17]. Ma et al. [18] discussed the effects of the ambient temperature on mortality in Wuhan, China. Liu et al. [19] and Xie and Zhu [20] discussed the effects of the ambient temperature and absolute humidity on the number of confirmed cases in Chinese cities. Tosepu et al. [21] discussed the effect of weather on the number of confirmed cases in Jakarta, Indonesia. Briz-Redón and Serrano-Aroca [22] performed a spatiotemporal analysis of temperature during the early evolution of COVID-19 in Spain. A similar analysis was conducted using data from Oslo, Norway [23]. Furthermore, recent studies have evaluated the effects of temperature and relative humidity on the morbidity rates in Brazil [24,25]. A numerical study on the spread of COVID-19 in Croatia has also been reported [26]. Studies using global data have discussed how temperature and humidity are correlated with the infection and fatality rates of COVID-19 [27,28]. However, the timeframe of the spread in each country may be different and difficult to define.

Our previous study [29] conducted with data from Japan suggested that the population density, which is somewhat indicative of social distancing, was more significant than the meteorological factors. The effect of population density on the morbidity rate was also discussed in a case study of Iran [30]. These studies suggested that several cofactors introduce uncertainty. When discussing the effects of policies, a multi-city analysis representing different countries may be imperative. In multi-country analyses, the number of conducted tests may add uncertainty because this number depends on medical resources and regional policies.

Unlike the morbidity and mortality rates, the duration of the pandemic may be less affected by different factors; e.g., it may not depend on the available medical resources. The understanding of the pandemic durations will be helpful for developing protection policies and governmental actions, to soften the potential damages to economy and social fabrics. In our recent study on 19 Japanese prefectures [31], the duration of the pandemic was discussed for the first time, and population density, absolute humidity, and temperature were found to be well-correlated in the spread stage. Conversely, the decay stage was predominantly affected by population density. The case study of COVID-19 in Japan provided insights into several environmental factors owing to the consistency and uniformity in data recording, healthcare quality, and social behavior. One open question is whether the findings obtained for one country can be applied globally.

The purpose of this novel study was to evaluate the effects of population density and meteorological factors on the pattern of spread and decay durations in cities or prefectures in four countries, including China, England, Germany, and Japan. To the best of our knowledge, a multi-country analysis focusing on the population, temperature and humidity factors of the COVID-19 duration has been hardly explored.

## 2 Material and methods

### 2.1 Study population

Data were collected for cities or prefectures from four countries, China, England, Germany and Japan, where medical resources collapse was not experienced (except for Wuhan, China). In our previous study [31], the correlation of population and meteorological factors with the spread and decay durations in Japan was studied. China is the first country reported the COVID-19 cases and imposed strict lockdown policies. England and German implemented restriction policies from March. The restrictions in all four countries have been eased before June. Moreover, the choice of the countries also depended on the data availability, the public accessibility of the statistics in city or prefecture level in the four countries allows the analyses to consider the effect of population and meteorological factors in fine resolution.

**Fig. 1:**
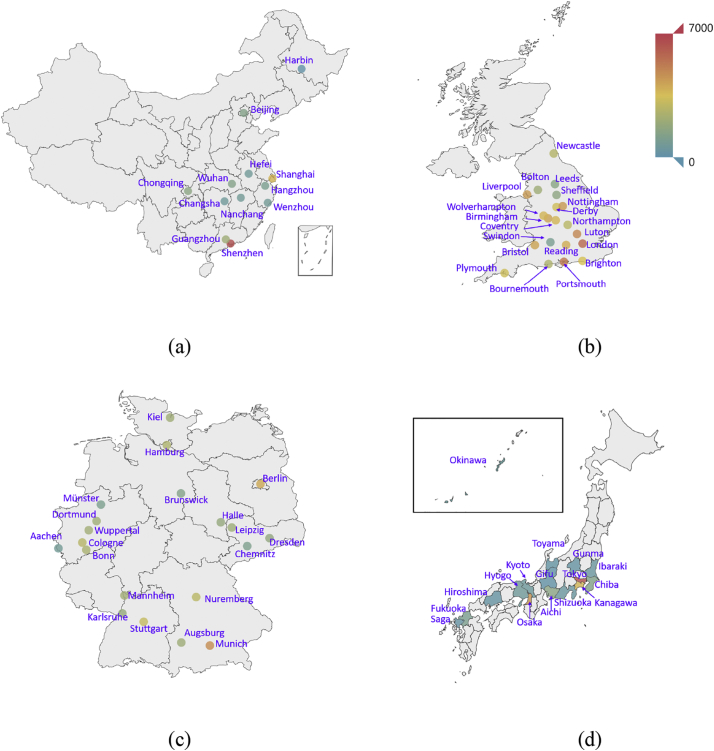
Map of studied cities or prefectures and population density (people per km^2^) in color scales in (a) China, (b) England, (c) Germany, and (d) Japan.

First, we selected the primate cities of each country, or those in which the pandemic began (Fig. 1). Wuhan was the first and most significant pandemic location in China. Data for municipalities directly under the central government and provincial capitals were collected. Those with a maximum daily increase in cases (7-day average) of less than 10 were excluded from the analysis. Wenzhou exhibited the largest daily increase outside Hubei Province, and it was also included. In England and Germany, we initially selected the cities with the highest population. However, several cities were excluded from the analysis if their daily case curves contained multiple peaks and did not decrease to 10% of the maximum 7-day average because the curves diverged from the bell-shaped model used. Finally, 20 cities in England and Germany were included in the analysis. In Japan, 16 prefectures had more than 10 confirmed daily positive cases; the data for these prefectures were included in the analysis.

### 2.2 Data sources

Three datasets were utilized in this study (shown in Table 1). The first dataset was the number of confirmed positive cases in each city. The data of the cities in China were collected from the dataset “China COVID-19 Daily Cases with Basemap” [32]. The data of the cities in England and Germany were collected from the referenced webpage [33] and NPGEO Corona Hub [34], respectively. The data of the prefectures in Japan were obtained from Toyo Keizai Online [35] and are based on a report by the Ministry of Health, Labour and Welfare [36]. The last date of data was June 23. Before June, the policies concerning the emergency in the four countries were eased. The number of confirmed positives may be influenced by the day of the week and latency, which is affected by the doctors' judgment. Therefore, we considered a moving average over 7 days (± 3 days in addition to the corresponding day) to reduce the effect of potential singularities.

**Table 1:** *P*: population (thousand persons); *PD*: population density (people per km^2^); *T*_ss_, *T*_se_, *D*_s_: starting date, termination date, and duration of the spread stage, respectively; *T*_ds_, *T*_de_, *D*_d_: starting date, termination date, and duration of the decay stage (days), respectively for the COVID-19 pandemic (2020) in different cities/prefectures in (a) China, (b) England, (c) Germany, and (d) Japan.

(a)	*P*	*PD*	*T*_ss_	*T*_se_	*D*_s_	*T*_ds_	*T*_de_	*D*_d_
Beijing	21,536	1312	17-Jan	28-Jan	12	2-Feb	17-Feb	16
Changsha	8155	690	22-Jan	30-Jan	9	4-Feb	16-Feb	13
Chongqing	8190	1260	20-Jan	30-Jan	11	4-Feb	21-Feb	18
Guangzhou	15,306	2059	23-Jan	30-Jan	8	5-Feb	14-Feb	10
Hangzhou	9806	582	21-Jan	29-Jan	9	1-Feb	14-Feb	14
Harbin	9515	179	24-Jan	2-Feb	10	10-Feb	20-Feb	11
Hefei	8087	707	21-Jan	4-Feb	15	7-Feb	17-Feb	11
Nanchang	5546	749	24-Jan	30-Jan	7	3-Feb	17-Feb	15
Shanghai	24,238	3823	16-Jan	26-Jan	11	1-Feb	14-Feb	14
Shenzhen	13,027	6484	22-Jan	30-Jan	9	4-Feb	16-Feb	13
Wenzhou	9300	768	23-Jan	29-Jan	7	5-Feb	14-Feb	10
Wuhan	11,081	1293	25-Jan	6-Feb	13	13-Feb	24-Feb	12

(b)	*P*	*PD*	*T*_ss_	*T*_se_	*D*_s_	*T*_ds_	*T*_de_	*D*_d_
Birmingham	1142	4264	14-Mar	27-Mar	14	3-Apr	25-May	53
Bolton	288	2057	23-Mar	5-Apr	14	13-Apr	23-May	41
Bournemouth	395	2451	22-Mar	6-Apr	16	16-Apr	12-May	27
Brighton	291	3514	15-Mar	3-Apr	20	16-Apr	8-May	23
Bristol	463	4213	15-Mar	7-Apr	24	18-Apr	13-May	26
Coventry	372	3766	8-Mar	28-Mar	14	5-Apr	22-May	39
Derby	257	3297	13-Mar	29-Mar	17	2-Apr	19-May	48
Leeds	793	1438	19-Mar	7-Apr	20	25-Apr	25-May	31
Liverpool	498	4455	18-Mar	3-Apr	17	9-Apr	18-May	40
London	8962	5712	7-Mar	25-Mar	19	4-Apr	6-May	33
Luton	213	4915	17-Mar	4-Apr	19	10-Apr	7-May	28
Newcastle	303	2631	17-Mar	31-Mar	15	10-Apr	10-May	31
Northampton	225	2781	19-Mar	1-Apr	14	8-Apr	22-Apr	15
Nottingham	333	4462	12-Mar	2-Apr	22	8-Apr	27-May	50
Plymouth	262	3283	15-Mar	11-Apr	28	23-Apr	21-May	29
Portsmouth	215	5339	12-Mar	5-Apr	25	13-Apr	9-May	27
Reading	162	4004	22-Mar	12-Apr	22	16-Apr	18-May	23
Sheffield	585	1590	15-Mar	27-Mar	13	4-Apr	17-May	44
Swindon	222	966	21-Mar	7-Apr	18	15-Apr	20-May	36
Wolverhampton	263	3793	9-Mar	18-Apr	41	27-Apr	6-Jun	41

(c)	*P*	*PD*	*T*_ss_	*T*_se_	*D*_s_	*T*_ds_	*T*_de_	*D*_d_
Aachen (District)	557	788	3-Mar	21-Mar	19	3-Apr	2-May	30
Augsburg	297	2020	12-Mar	24-Mar	13	29-Mar	30-Apr	33
Berlin	3669	4118	9-Mar	23-Mar	15	1-Apr	24-May	54
Bonn	330	2337	8-Mar	21-Mar	14	5-Apr	9-May	35
Brunswick	249	1298	16-Mar	28-Mar	13	1-Apr	18-Apr	18
Chemnitz	246	1115	13-Mar	28-Mar	16	31-Mar	19-Apr	20
Cologne	1088	2685	9-Mar	16-Mar	8	1-Apr	6-May	36
Dortmund	588	2096	13-Mar	3-Apr	22	5-Apr	8-May	34
Dresden	557	1693	10-Mar	22-Mar	13	30-Mar	8-May	40
Halle (Saale)	239	1768	13-Mar	26-Mar	14	1-Apr	2-May	32
Hamburg	1847	2446	9-Mar	20-Mar	12	28-Mar	2-May	36
Karlsruhe	312	1799	10-Mar	24-Mar	15	6-Apr	10-May	35
Kiel	247	2081	13-Mar	25-Mar	13	1-Apr	7-May	37
Leipzig	593	1995	9-Mar	21-Mar	13	3-Apr	11-May	39
Mannheim	311	2143	9-Mar	24-Mar	16	6-Apr	5-May	30
Munich	1484	4781	11-Mar	26-Mar	16	31-Mar	14-May	45
Münster	315	1041	12-Mar	17-Mar	6	26-Mar	16-Apr	22
Nuremberg	518	2780	15-Mar	3-Apr	20	8-Apr	9-May	32
Stuttgart	636	3067	8-Mar	16-Mar	9	30-Mar	18-May	50
Wuppertal	355	2109	14-Mar	4-Apr	22	10-Apr	17-May	38

(d)	*P*	*PD*	*T*_ss_	*T*_se_	*D*_s_	*T*_ds_	*T*_de_	*D*_d_
Aichi	7552	1460	22-Feb	30-Mar	38	1-Apr	27-Apr	27
Chiba	6259	1217	19-Mar	2-Apr	15	13-Apr	5-May	23
Fukuoka	5104	1025	22-Mar	1-Apr	11	9-Apr	27-Apr	19
Gifu	1987	187	25-Mar	4-Apr	11	6-Apr	17-Apr	12
Gunma	1942	305	25-Mar	5-Apr	12	9-Apr	22-Apr	14
Hiroshima	2804	331	26-Mar	6-Apr	12	10-Apr	27-Apr	18
Hyogo	5466	650	19-Mar	4-Apr	17	7-Apr	4-May	28
Ibaraki	2860	470	16-Mar	28-Mar	13	8-Apr	23-Apr	16
Kanagawa	9198	3808	19-Mar	3-Apr	16	11-Apr	19-May	39
Kyoto	2583	560	16-Mar	2-Apr	18	5-Apr	9-May	35
Okinawa	1453	638	28-Mar	3-Apr	7	10-Apr	25-Apr	16
Osaka	8809	4631	18-Mar	6-Apr	20	13-Apr	6-May	24
Saga	815	334	23-Mar	15-Apr	24	22-Apr	1-May	10
Shizuoka	3644	468	25-Mar	3-Apr	10	6-Apr	27-Apr	22
Tokyo	13,921	6355	17-Mar	3-Apr	18	10-Apr	7-May	28
Toyama	1044	246	1-Apr	13-Apr	13	18-Apr	30-Apr	13

The second dataset was population and population density in each city, as shown in Table 1. The population data of China were collected from the Statistical Yearbook for each municipality or province. The population data of England were collected from the Office for National Statistics [37]. The population data of Germany were collected from the Statistical Offices of the Federal Government and the Federal States [38]. The data of the prefectures in Japan were obtained from the Statistics Bureau of Japan [39].

The third dataset focused on weather information. The maximum and minimum temperature and relative humidity in China, England, and Germany were obtained from the National Centers for Environmental Information [40]. The weather data of each Japan prefecture were obtained from the Japan Meteorological Agency [41] during the time of the pandemic. The absolute humidity was derived from the relative humidity and ambient temperature data. The meteorological factors for the selected cities are shown in Table S1.

### 2.3 Outcome variables

**Fig. 2:**
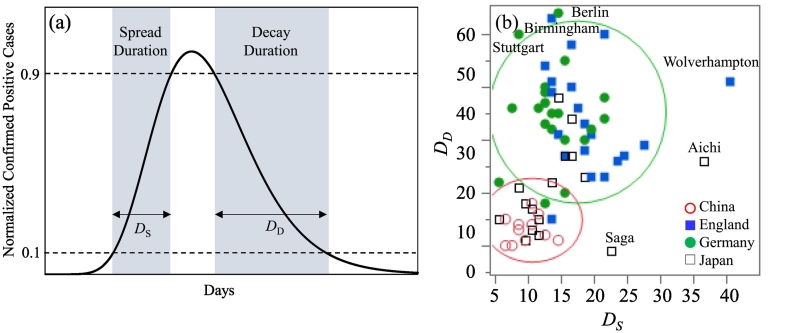
(a) Definition of the spread (*D*_*S*_) and decay durations (*D*_*D*_) in days along the curve of daily normalized confirmed positive cases of COVID-19 [31]. (b) Relationship between *D*_*S*_ and *D*_*D*_ in days. The red and green ellipses mainly contain cities in China and Japan and those in England and Germany. (For interpretation of the references to color in this figure legend, the reader is referred to the web version of this article.)

In our previous study [31], the number of days required for spreading from 10 to 90% and those for decaying from 90% to 10% of the peak of the confirmed positives (7-day average), namely the spread duration (*D*_*S*_) and decay duration (*D*_*D*_), were established as metrics for evaluation of the time span of the pandemic outbreak. An illustration of the definition of the spread and decay durations are shown in Fig. 2 a). Fig. 2 b) shows the relationship between the two durations, *K*-means clustering (*n* = 2) was first applied to separate the data into two major groups. The red group mainly contained cities in China and Japan, whereas the green group contained cities in England and Germany. In the latter clustering, Birmingham and Wolverhampton (England), Berlin and Stuttgart (Germany), and Aichi and Saga (Japan) were excluded as outliers because they were not in the clustering circle. This tendency might have been caused by an overlap of repetitive smaller waves or cluster infections, which do not follow the theoretical spread curves. For example, dual peaks were observed in Aichi during the spread and decay stages.

### 2.4 Statistical analysis

Statistical analysis was then conducted to determine the correlation between different factors and the spread and decay durations. A multivariable analysis using linear regression was then conducted with the following model:

(1) $$y=\beta_{0}+\beta_{1}\cdot \mathrm{PD}+\beta_{2}\cdot \mathrm{AH}+\beta_{3}\cdot T_{\min}+\beta_{4}\cdot T_{\max}+\beta_{5}\cdot T_{\mathrm{avg}}+\epsilon$$

where *β*_0_ is intercept, *β*_1_ − *β*_5_ are regression coefficients, *ϵ* is residual, *y* is the output variable i.e. *D*_*S*_ or *D*_*D*_. In the equation, *y* linearly depends on a combination of independent variables of population density and meteorological factors. Statistical significance was accepted at *p* < 0.05, with test of null hypothesis that *β*_*i*_ = 0, *i* = 1, …, 5.

## 3 Results

**Fig. 3:**
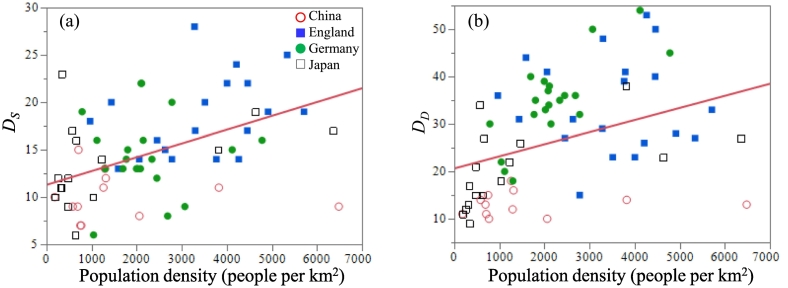
Relationship of (a) spread and (b) decay durations (*D*_*S*_ and *D*_*D*_, respectively) with population density.

Fig. 3 shows the dependence of the spread and decay durations on the population density (also see Table S2). The overall correlation of the spread durations with the population densities of four countries was statistically significant (*p* < 0.0001); however, only Japan exhibited a statistical significance *(p* < 0.05) when each country was considered individually. A similar tendency was observed for the decay duration; the correlation with the population density was statistically significant (*p* < 0.0001) when data from all countries were included in the evaluation. However, only Germany (*p* < 0.005) exhibited statistically significant correlations. The durations of the spread and decay stages were not influenced by the population density in China.

**Fig. 4:**
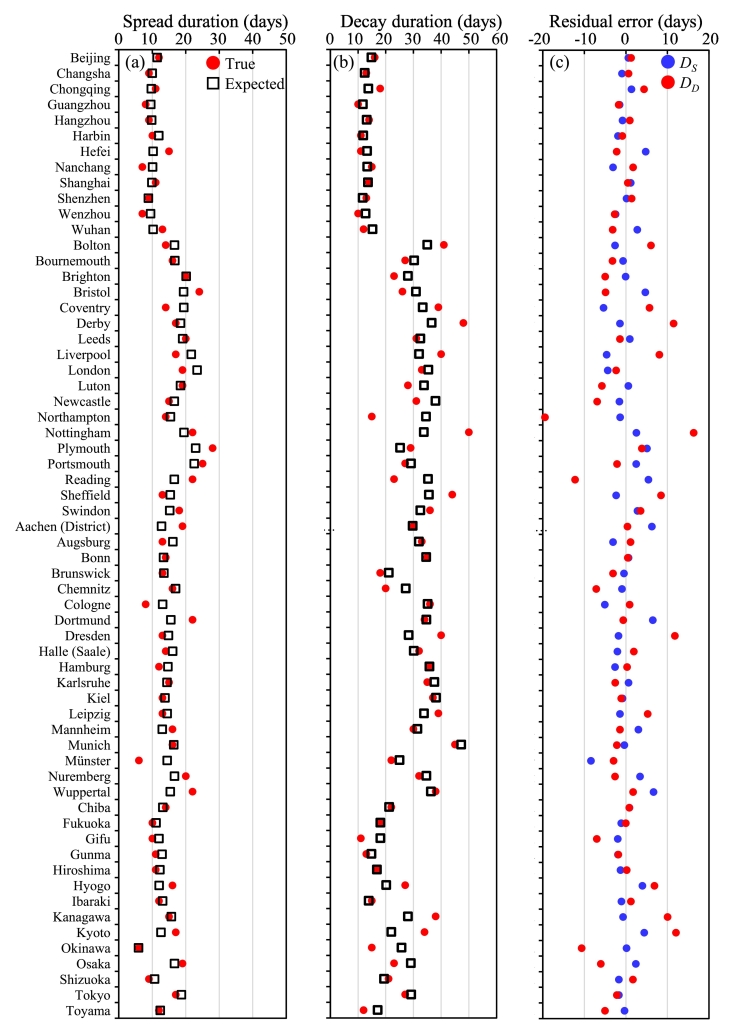
Multivariable analysis with the variables listed in Table 2(b) for each country. The number of days of (a) spread duration, (b) decay duration, and (c) residual error.

A multivariable analysis was conducted to investigate the relationship between the spread and decay durations of the pandemic with the absolute humidity and ambient temperature measured during the corresponding periods, as well as with the population density. As shown in Fig. 4 and Table 2, the duration of the spread period was characterized with respect to population density and absolute humidity, and the decay period was characterized with respect to population density, absolute humidity, and daily maximum temperature (*p* < 0.0001 for both the spread and decay durations). The maximum temperature was related to the maximum absolute humidity. Thus, multicollinearity was evaluated based on the variance inflation factors (VIFs). We used the threshold value of the VIF to differentiate between low and high contributions, which is commonly taken as 10 [42]. The higher the maximum temperature and absolute humidity, the shorter the spread and decay periods in the analysis of the four countries. The contributions of the population density were 64% and 28% in the spread and decay durations, respectively, and the remaining proportions were explained by the absolute humidity and temperature.

**Table 2:** Multivariable analysis for spread (*D*_*S*_) and decay (*D*_*D*_) durations for (a) all countries and (b) individual countries. *PD* represents the population density. *T*_*ave*_, *T*_*max*_, and *T*_*min*_ represent the daily average, maximum, and minimum temperatures, respectively. *AH* represents the daily average absolute humidity values.

(a)	Variables	*R*^2^	adj. *R*^2^	*p*-value	VIF	Residual
*D*_*S*_	PD, AH	0.32	0.30	<0.0001	1.00	2.77
*D*_*D*_	PD, *T*_*max*_, AH	0.35	0.32	<0.0001	1.74	6.60

(b)	Variables	*R*^2^	adj. *R*^2^	*p*-value	VIF	Residual
China						
*D*_*S*_	AH	0.12	0.03	0.27	1.00	1.79
*D*_*D*_	*T*_*max*_, AH	0.21	0.03	0.35	7.88	1.78
England						
*D*_*S*_	PD, *T*_*min*_	0.39	0.30	<0.05	1.12	2.45
*D*_*D*_	AH	0.12	0.06	0.16	1.00	6.34
Germany						
*D*_*S*_	*T*_*min*_	0.10	0.04	0.20	1.00	2.71
*D*_*D*_	PD*, T*_*ave*_, AH	0.68	0.61	<0.001	2.44	2.37
Japan						
*D*_*S*_	PD, AH	0.66	0.60	<0.005	1.02	1.47
*D*_*D*_	PD, *T*_*max*_	0.40	0.29	0.06	1.20	4.10

The decay duration in Germany exhibited statistical significances with population density, average temperature, and absolute humidity, the spread duration in England with population density and daily minimum temperature, and the spread period in Japan with population density and absolute humidity. The *p*-value of the decay period in Japan with respect to the daily maximum temperature was 0.063. As listed in Table 2, the residual spread and decay durations were 2.77 days and 6.60 days for all four countries, whereas the average residuals were 2.18 days and 3.84 days for the individual countries, respectively. The effect of population density was nonexistent in China, as shown in Fig. 3.

## 4 Discussion and conclusion

Understanding the potential risks during the pandemic is essential for managing medical resources. Furthermore, a model that can be used to estimate the potential spread and decay durations of the pandemic enables the implementation of evidence-based government policies. This may reduce the potential damage to the economy, education, and various daily activities that have suffered during the lockdown restrictions. This study presented a data analysis of how meteorological factors and population density are correlated to the spread and decay durations of the COVID-19 pandemic in four countries, in some cities of which, the first wave has already decayed. Specifically, both durations exhibited a high correlation with the population density. Longer durations were expected in cities with higher population densities, implicitly suggesting the importance of social distancing [43]. This has been confirmed in cities of England and Germany, which was observed in Japan [31]. The opposite tendency found in China can be explained by the fact that the strict lockdown imposed social distancing despite the high population density.

Direct evaluation of the polices across different countries is difficult, owing to the differences in the testing rates, public response, culture etc. From the collected dataset and the analysis, the effect of policy may be implicitly inferred. In China, short durations and insignificant correlations of durations with population and meteorological factors were observed owing to the broadly strict lockdown policies. In England, mixed signals (such as “herd immunity”) was sent out in the beginning, and criticism appeared for the government of not acting quickly in March. In Germany, in the beginning of the pandemic, the government responded with actions such as contact tracing and testing, followed by prohibition of gathering with strict punishment for violation. The multivariable regression analysis revealed significant correlations between spread duration and factors in UK, but not in Germany. The opposite trend observed for decay duration. In Japan, unlike many other countries where city lockdowns were enforced, citizens self-isolated during this state of emergency; stronger correlations for spread and decay durations observed. From these finding, it may be suggested that the strict restriction and its implementation may be related to less correlation of the durations of the pandemic waves with the population and meteorological factors.

Both temperature and absolute humidity were less correlated with the durations than with the population density. This study conducted a multivariable analysis considering population density, maximum and minimum temperature, and absolute humidity. The adjusted *R*^2^ values were 0.30 and 0.32 for the durations of the spread and decay stages, respectively, and they were statistically significant. The spreading and decay durations were sufficiently estimated from the multivariable analysis; the residual spread and decay durations were 2.77 days and 6.60 days for all countries, whereas the average residuals were 2.18 days and 3.84 days for the individual countries. This suggested that we can estimate the durations based on these parameters. One limitation of the analysis in this study is that the daily-increase curves in some cities diverge from the bell-shape used for defining the spread and decay durations, owing to the repetitive sub waves and cluster infections.

In conclusion, the durations of the spread and decay stages of the COVID-19 pandemic were correlated with absolute humidity, temperature, and population density in four countries that did not experience a collapse of their medical systems. The contributions of these factors were different in each country, which could be attributed to regional policies. Although the morbidity and mortality rates differed by one to two orders of magnitude, these parameters were sufficient to estimate the durations. The higher population density tends to lead to prolonged spread and decay durations, while less correlations with the population and meteorological factors. The contribution rate of population density for multivariable are 30–50% in each country. The tendency of shortening duration with higher temperature and humidity reported by [31] was not clearly observed in Germany and England. These durations were affected by the policies and human behavior. With strict lockdown policies imposed (China), durations are shortened and their correlations with the population density and meteorological factor become insignificant. These findings will be useful in policy setting and governmental actions, such as determining the necessary shutdown length. This factor should be considered in another potential pandemic or next waves of COVID-19, in addition to the results of the multi-city comparison.

## Funding

The authors declare that we have no financial support for the research, authorship, and/or publication of this article.

## Authors' contributions

Conceptualization: YD, AH & ER. Data curation: YD, DA, SK, Formal analysis: YD, DA & SK. Investigation: SK, JG & AH. Methodology: YD, SK, ER & AH. Project administration: AH & ER. Resources. Software. Supervision: AH & ER. Validation: YD, DA & SK. Visualization: SK & YD. Roles/Writing: DA, YD, ER & AH. Writing, review & editing: All.

## Declaration of Competing Interest

The authors declare no competing interests.

## Appendix A Policies in different countries

The rapid spread of the virus in China in early 2020 lead several countries to adopt drastic measures at different scales. China [44] enforced a strict lockdown, which started in Wuhan and expanded to the whole province. On February 10, Beijing and Shanghai, two of China's largest cities, restricted the movement of residents and vehicles, made wearing masks compulsory, and shut down leisure and other non-essential community services [45].

The first transmission of COVID-19 in England was confirmed in February. Initially, the government decided not to shut down large gatherings or introduce restrictions. On March 20, the Prime Minister announced in a television broadcast that all pubs, cafes, restaurants, bars, and gyms were to close. On March 23, a nationwide lockdown was announced. At this point, there were 2433 confirmed cases in London. The closure of pubs, restaurants, and indoor sports and leisure facilities was imposed via the Health Protection (Coronavirus, Business Closure) (England) Regulations 2020 [46]. On May 10, plans to ease lockdown restrictions were announced [47].

In Germany, authorities shut down schools and stopped major sports events on March 13, 2020, which was closely followed by further decisions regarding restaurants, shops, and other services [48]. The government banned gatherings in churches, mosques, and synagogues, and ordered the closure of non-essential shops, playgrounds, and schools. Moreover, regional trains were curtailed in an effort to reduce travel. On May 6, Chancellor announced steps to ease the coronavirus lockdown in Germany [49].

In Japan, a state of emergency was declared nationwide on April 16 after being implemented to only seven prefectures on April 7 [50]. During this state of emergency, people self-contained voluntarily instead of a government-enforced lockdown of the prefectures. This state of emergency was discontinued on May 23.

## Appendix B Supplementary data

Supplementary material Image 1
