# Supplementary material for: Influence of population density, temperature, and absolute humidity on spread and decay durations of COVID-19: A comparative study of scenarios in China, England, Germany, and Japan
Source: One Health. 2020 Dec 11;12:100203. doi: 10.1016/j.onehlt.2020.100203 (PMC7736723; doi:10.1016/j.onehlt.2020.100203)
Supplement: Supplementary file 1 — Supplementary material [file mmc1.docx]

Table S1. Daily average, maximum and minimum temperature, and daily average absolute humidity in (a) China, (b) England, (c) Germany, and (d) Japan. The values are averaged during the spread and decay stages as listed in Table 1. *T_ave_*, *T_max_*, and *T_min_* represent the daily average, maximum, and minimum temperatures, respectively. *AH* represents the daily average absolute humidity values.

| (a) | Spread duration | | | | Decay duration | | | |
| --- | --- | --- | --- | --- | --- | --- | --- | --- |
|  | *T_ave_* | *T_max_* | *T_min_* | *AH* | *T_ave_* | *T_max_* | *T_min_* | *AH* |
| Beijing | -1.5 | 6.1 | -8.6 | 2.2 | -1.2 | 6.4 | -7.2 | 2.7 |
| Changsha | 5.8 | 9.1 | 2.9 | 6.2 | 9.7 | 13.6 | 6.2 | 7.8 |
| Chongqing | 9.1 | 13.3 | 6.5 | 7.0 | 10.3 | 15.0 | 6.7 | 7.1 |
| Guangzhou | 14.3 | 21.0 | 9.2 | 7.4 | 16.8 | 20.0 | 11.7 | 10.9 |
| Hangzhou | 7.4 | 9.6 | 4.9 | 6.6 | 9.5 | 13.7 | 5.3 | 7.2 |
| Harbin | -16.9 | -8.6 | -25.0 | 0.9 | -9.1 | -2.6 | -16.1 | 1.8 |
| Hefei | 4.9 | 9.8 | -0.7 | 5.5 | 6.8 | 11.9 | -0.3 | 6.3 |
| Nanchang | 5.8 | 9.2 | 3.3 | 5.8 | 9.8 | 14.4 | 6.5 | 7.2 |
| Shanghai | 7.0 | 9.9 | 4.1 | 6.6 | 8.4 | 13.2 | 3.0 | 6.6 |
| Shenzhen | 16.5 | 22.1 | 12.8 | 9.3 | 17.5 | 22.2 | 14.5 | 11.8 |
| Wenzhou | 10.9 | 15.0 | 8.9 | 7.5 | 11.7 | 15.8 | 8.1 | 8.3 |
| Wuhan | 6.4 | 12.0 | 0.2 | 5.4 | 10.6 | 16.9 | 2.4 | 6.6 |

| (b) | Spread duration | | | | Decay duration | | | |
| --- | --- | --- | --- | --- | --- | --- | --- | --- |
|  | *T_ave_* | *T_max_* | *T_min_* | *AH* | *T_ave_* | *T_max_* | *T_min_* | *AH* |
| Birmingham | 6.8 | 11.4 | 2.4 | 5.5 | 11.6 | 16.8 | 5.7 | 6.5 |
| Bolton | 7.6 | 12.5 | 2.2 | 5.3 | 10.7 | 16.2 | 4.4 | 6.5 |
| Bournemouth | 7.6 | 13.1 | 2.0 | 5.3 | 11.5 | 17.1 | 5.5 | 7.1 |
| Brighton | 7.3 | 11.0 | 3.5 | 5.7 | 11.0 | 15.6 | 6.0 | 7.4 |
| Bristol | 7.0 | 11.2 | 2.6 | 5.6 | 10.8 | 15.6 | 5.9 | 7.1 |
| Coventry | 7.0 | 11.2 | 2.9 | 5.8 | 11.2 | 16.8 | 5.2 | 6.7 |
| Derby | 6.4 | 10.7 | 2.6 | 5.3 | 10.6 | 15.8 | 5.3 | 6.3 |
| Leeds | 7.7 | 11.0 | 4.0 | 5.3 | 11.8 | 13.9 | 9.1 | 6.9 |
| Liverpool | 7.9 | 12.3 | 3.9 | 5.6 | 11.5 | 16.6 | 6.7 | 6.9 |
| London | 8.5 | 12.6 | 4.3 | 6.0 | 12.2 | 18.2 | 6.5 | 6.5 |
| Luton | 6.0 | 10.6 | 1.7 | 5.2 | 10.2 | 16.0 | 4.7 | 6.7 |
| Newcastle | 6.0 | 10.1 | 1.8 | 5.5 | 8.7 | 13.4 | 3.5 | 6.1 |
| Northampton | 5.3 | 10.3 | 1.0 | 4.9 | 11.0 | 17.3 | 5.3 | 6.6 |
| Nottingham | 6.4 | 10.4 | 2.6 | 5.3 | 11.4 | 16.6 | 6.2 | 6.7 |
| Plymouth | 9.3 | 13.3 | 5.4 | 6.4 | 11.8 | 16.3 | 7.4 | 7.8 |
| Portsmouth | 7.6 | 11.2 | 4.0 | 5.8 | 11.0 | 15.4 | 6.3 | 7.3 |
| Reading | 8.6 | 15.4 | 1.1 | 5.3 | 11.0 | 16.9 | 4.3 | 6.5 |
| Sheffield | 6.9 | 12.2 | 1.6 | 0.6 | 10.2 | 15.9 | 4.3 | 6.4 |
| Swindon | 7.1 | 12.1 | 1.9 | 5.0 | 11.0 | 16.2 | 5.6 | 6.9 |
| Wolverhampton | 8.5 | 13.1 | 3.6 | 5.8 | 12.9 | 17.8 | 7.5 | 7.0 |

| (c) | Spread duration | | | | | Decay duration | | | |
| --- | --- | --- | --- | --- | --- | --- | --- | --- | --- |
|  | *T_ave_* | *T_max_* | *T_min_* | *AH* | *T_ave_* | | *T_max_* | *T_min_* | *AH* |
| Aachen (District) | 8.9 | 11.4 | 6.2 | 6.9 | 12.6 | | 18.8 | 6.1 | 6.2 |
| Augsburg | 5.7 | 12.0 | 0.1 | 4.9 | 9.5 | | 16.9 | 1.6 | 5.3 |
| Berlin | 7.1 | 11.2 | 2.6 | 4.9 | 11.8 | | 17.4 | 5.2 | 5.7 |
| Bonn | 9.5 | 13.8 | 5.1 | 6.4 | 13.1 | | 20.0 | 5.3 | 6.1 |
| Brunswick | 8.7 | 11.3 | 4.9 | 4.0 | 11.9 | | 15.3 | 6.9 | 4.8 |
| Chemnitz | 5.2 | 10.9 | -1.4 | 4.2 | 8.1 | | 15.1 | 0.6 | 4.9 |
| Cologne | 9.4 | 13.3 | 5.6 | 6.6 | 12.2 | | 18.9 | 4.5 | 5.8 |
| Dortmund | 6.3 | 10.9 | 1.2 | 4.5 | 12.1 | | 18.1 | 5.6 | 6.1 |
| Dresden | 6.7 | 10.9 | 2.5 | 5.0 | 10.3 | | 15.7 | 4.3 | 5.2 |
| Halle (Saale) | 5.2 | 10.3 | 0.2 | 4.3 | 10.6 | | 16.9 | 3.9 | 5.4 |
| Hamburg | 7.5 | 11.1 | 2.8 | 5.9 | 9.0 | | 14.7 | 2.9 | 5.4 |
| Karlsruhe | 8.6 | 14.2 | 3.3 | 5.9 | 14.1 | | 21.5 | 6.5 | 7.0 |
| Kiel | 6.7 | 8.6 | 4.3 | 3.7 | 10.0 | | 13.1 | 6.2 | 6.1 |
| Leipzig | 7.7 | 12.2 | 3.2 | 5.8 | 11.1 | | 17.3 | 4.4 | 5.8 |
| Mannheim | 10.4 | 13.7 | 5.8 | 5.7 | 16.3 | | 20.5 | 10.6 | 6.5 |
| Munich | 5.5 | 11.4 | -0.4 | 4.7 | 10.5 | | 17.4 | 2.7 | 5.9 |
| Münster | 8.5 | 12.5 | 3.2 | 5.7 | 9.1 | | 15.1 | 2.2 | 4.9 |
| Nuremberg | 5.7 | 12.0 | -0.8 | 3.9 | 12.4 | | 19.7 | 4.4 | 5.8 |
| Stuttgart | 7.7 | 13.2 | 2.7 | 5.7 | 11.7 | | 18.3 | 4.6 | 5.9 |
| Wuppertal | 7.2 | 12.3 | 1.5 | 4.6 | 12.2 | | 18.5 | 5.0 | 6.3 |

| (d) | Spread duration | | | | Decay duration | | | |
| --- | --- | --- | --- | --- | --- | --- | --- | --- |
|  | *T_ave_* | *T_max_* | *T_min_* | *AH* | *T_ave_* | *T_max_* | *T_min_* | *AH* |
| Aichi | 10.1 | 14.8 | 6.0 | 5.9 | 13.0 | 18.3 | 8.6 | 6.5 |
| Chiba | 12.4 | 16.1 | 8.1 | 6.6 | 15.1 | 19.1 | 11.2 | 8.4 |
| Fukuoka | 14.2 | 17.5 | 11.3 | 8.8 | 14.0 | 17.5 | 10.9 | 7.3 |
| Gifu | 12.0 | 16.4 | 7.7 | 6.7 | 12.6 | 18.2 | 7.7 | 5.1 |
| Gunma | 10.6 | 15.3 | 5.4 | 5.7 | 11.5 | 16.3 | 7.2 | 6.3 |
| Hiroshima | 12.4 | 16.2 | 8.6 | 6.5 | 13.2 | 17.4 | 9.2 | 5.6 |
| Hyogo | 12.7 | 16.4 | 9.1 | 7.2 | 15.5 | 19.0 | 12.4 | 8.1 |
| Ibaraki | 10.3 | 17.1 | 3.4 | 5.7 | 10.8 | 15.6 | 6.4 | 6.5 |
| Kanagawa | 12.4 | 16.7 | 8.0 | 6.8 | 16.6 | 20.7 | 13.0 | 9.8 |
| Kyoto | 11.5 | 16.6 | 6.8 | 6.4 | 14.7 | 20.1 | 10.0 | 7.1 |
| Okinawa | 21.3 | 24.0 | 18.8 | 14.7 | 19.8 | 22.1 | 17.6 | 11.8 |
| Osaka | 12.7 | 17.0 | 8.9 | 6.7 | 16.2 | 20.6 | 12.3 | 8.1 |
| Saga | 13.4 | 17.9 | 9.0 | 7.3 | 14.9 | 20.1 | 9.8 | 7.1 |
| Shizuoka | 13.1 | 16.6 | 9.3 | 8.6 | 14.3 | 18.7 | 10.0 | 7.1 |
| Tokyo | 11.7 | 16.7 | 6.7 | 6.4 | 14.4 | 19.2 | 9.9 | 8.6 |
| Toyama | 9.7 | 14.6 | 5.2 | 6.3 | 12.1 | 17.6 | 7.7 | 7.5 |

Table S2. Coefficients of determination *R*^2^, adjusted *R*^2^ values and *p*-values for relationship of spread and decay duration with population density in four countries and each country.

| Country | Spread Duration | | | Decay Duration | | |
| --- | --- | --- | --- | --- | --- | --- |
|  | *R*^2^ | adj. *R*^2^ | *p*-value | *R*^2^ | adj. *R*^2^ | *p*-value |
| Four countries | 0.24 | 0.23 | <0.0001 | 0.26 | 0.25 | <0.0001 |
| China | 0.00 | -0.10 | 0.85 | 0.003 | -0.10 | 0.85 |
| England | 0.19 | 0.14 | 0.07 | 0.01 | -0.05 | 0.70 |
| Germany | 0.01 | -0.05 | 0.66 | 0.47 | 0.44 | <0.005 |
| Japan | 0.40 | 0.35 | <0.05 | 0.27 | 0.21 | 0.06 |
